# Event-related evoked potential versus clinical tests in assessment of subclinical cognitive impairment in chronic hepatitis C virus

**DOI:** 10.1186/s41983-018-0034-y

**Published:** 2018-11-20

**Authors:** Hanaa Khalaf Fath-Elbab, Elham Ahmed, Dina Fathy Mansour, Wail Talaat Soliman

**Affiliations:** Minia University Faculty of Medicine, Minia, Egypt

**Keywords:** Chronic hepatitis C, Neurocognitive function, P300, Liver fibrosis

## Abstract

**Context:**

Chronic infection by hepatitis C virus causes impairment in neurocognitive function in up to 50% of patients which may not be detected by clinical tests.

**Aim:**

Early detection of neurocognitive impairment in chronic hepatitis C patients and investigating the cognitive function in HCV patient by p300 and clinical test.

**Materials and methods:**

The study included 60 patients with chronic hepatitis C and 30 healthy controls. Participants were subjected to a biochemical, hematological assessment, mini-mental state examination, Montreal Cognitive Assessment, P300, polymerase chain reaction (PCR), and fibroscan made for hepatitis C patients.

**Results:**

The digit span, attention, concentration, and memory were significantly lower in patients than controls. The delayed P300 peak latency and the reduction of its amplitude were significantly evident in patients with liver fibrosis than the controls and patients without fibrosis. These abnormalities were significantly higher with increasing the grade of fibrosis. All patients with cognitive impairment (reduced mini-mental state score) had abnormal P300-evoked responses. P300 could detect neurocognitive impairment in some patients with normal neurocognitive functions by clinical test. P300 had sensitivity (100%), specificity (59.26), positive predictive value (75%), negative predictive value (100%), and accuracy (81.67) in the detection of neurocognitive impairment in HCV patient.

**Conclusion:**

Patients with chronic hepatitis C infection had significant impairment in their cognitive functions. This impairment increases with the increase in grade of hepatic fibrosis. P300 can detect minimal and subclinical impairment of cognitive function at early stages of chronic hepatitis with accuracy (81.67).

**Trial registration:**

PACTR on 19 march 2018 retrospectively. Identification number for the registry is PACTR201804003215168.

## Key messages

Impairment of cognitive functions increases with the increase in grade of hepatic fibrosis in HCV patients. P300 can detect minimal and subclinical impairment of cognitive function at early stages of chronic hepatitis.

## Introduction

Hepatitis C virus (HCV) infection is a serious global health problem that affects 180 million people worldwide. Hepatitis C virus causes acute and chronic hepatitis which can eventually lead to permanent liver damage and hepatocellular carcinoma [1]. HCV infection is considered a systemic disease. Symptoms of brain dysfunction such as fatigue and depression are frequent in patients with chronic hepatitis C than in chronic hepatitis B, and these symptoms improved after clearing the virus with treatment [2]. HCV is neurovirulent. The exact mechanism by which HCV enters the central nervous system is unknown; HCV may cross the blood–brain barrier via infected monocytes using a Trojan horse mechanism [3]. Neuroimaging studies suggest that HCV-positive individuals show altered structure and function of several neuronal systems, including the frontal neocortex, basal ganglia, and connecting white matter tracts [3]. Neurocognitive disorders have been reported in up to 50% of chronic HCV infected patients [4]. HCV patients had significant deficits in attention, learning, psychomotor speed, and mental flexibility; greater impairment was associated with greater fibrosis stage [5]. Forton et al. [6] compared cognitive function in individuals with HCV viremia and those with only anti-HCV antibodies. He found that HCV-infected patients were impaired on more cognitive tasks than the HCV-cleared group and viremic individuals showed specific impairment in power of concentration and speed of working memory [6]. Cognitive deficits may be found in HCV (hepatitis c virus) patients even prior to the development of cirrhosis and liver dysfunction [2]. P300-evoked response is an objective and independent measure of cerebral information processing to avoid the potential bias of fatigue, latent depression, or impaired self-rating in psychometric assessment [7].

This study aimed at early detection of neurocognitive impairment in chronic hepatitis C patients and investigating the cognitive function in HCV patient by p300 and clinical test.

### Subjects and methods

The study included 60 untreated chronic hepatitis C patients in addition to 30 healthy persons matched for age, sex, and educational level. The majority of participants achieved a high level of education (secondary school to university). All subjects did not give a history of cognitive dysfunction. We excluded patients with liver cirrhosis, other causes of chronic liver diseases rather than HCV, psychiatric disorders patients, patients with conditions that limit the fibroscan technique (such as ascites), obese patients with BMI ≥ 28, and patients with renal failure, diabetes, or history of drug abuse in the last 6 months. Fibroscan was done in HCV patients to detect the grade of liver fibrosis. The HCV group was subdivided into three groups according to the grade of liver fibrosis (F0–F1, F2, F3). A written informed consent was taken from patients and control before participation according to the guidelines of 1975 declaration of Helsinki. The patients were randomly selected from the outpatient clinic of the tropical medicine department and internal medicine department Minia University Hospital. The controls were randomly selected from outpatient clinic and relatives of the patients admitted. Both patients and control groups were subjected to the following clinical assessment and laboratory investigations, especially liver and kidney functions, blood sugar, HCV-AB (antibodies), HCV PCR, HIV (human immune deficiency virus), and abdominal ultrasound.

Regarding FibroScan, we used M probe for measuring liver stiffness. The device generates a 50-Hz shear wave. The patients were in a supine position. Transducer probe was placed on the skin, between the rib bones at the level of the right lobe of the liver. The patient was holding breath and the operator pressed the probe button to take the measurements. Ten measurements were taken to detect liver stiffness which was expressed in kilopascals (kPa) [8]. Cut-off values were 7.1 kPa for F ≥ 2, 9.5 kPa for F ≥ 3, and 12.5 kPa for F = 4 [9].

Montreal cognitive assessment scores (MoCA) version A [10] was done to all patients and control to detect the mean of digit span, attention, concentration, memory, orientation, and total scores. Total mini-mental state examination (MMSE) scores were calculated as follows: 25–30 was considered normal, 20–24 indicates mild cognitive impairment, 11–19 indicates moderate cognitive impairment, and a score of 10 or less indicates severe cognitive impairment [11].

Regarding P300, oddball task auditory-evoked potentials (peak of p300 latency) using a Nihon Kohden Neuropack MEB-9204 was done. The electrophysiological activity was recorded from three different positions: Fz (forehead midline), Cz (coronal midline), and Pz (parietal midline). Impedances were maintained between 5 and 10 kΩ. Artifacts were automatically rejected at ± 50 μV. The stimuli were presented through headphones at 90 dispel in both ears. The subjects were seated in a comfortable chair, quiet and properly illuminated room, awake and exposed to a series of standard auditory stimuli and small amounts (15 or 20%) of deviant stimuli. Subjects were asked to detect or count the target stimuli (active task) reporting the total at the end of the session. The test was initiated only when the subject demonstrated a complete understanding of the task. The P300 component was defined as the largest positive-going peak occurring as a reaction for the deviant (rare) stimuli within a specific latency window (250–450 ms). Peak amplitude was measured relative to the prestimulus baseline (100 ms), and peak latency was measured from the time of stimulus onset [12].

All analyses were performed with version 20 of Statistical Package of Social Science (SPSS). Qualitative data were expressed as proportions, while quantitative data were expressed as mean + standard deviation (SD). Unpaired Student’s *t* test was used to compare data between two groups for normally distributed data, while ANOVA was used to compare data between the four groups. Qualitative data were analyzed by chi-square (*χ*^2^) test. Statistical significance was defined as *p* values less than 0.05.

We calculated sensitivity, specificity, positive predictive value (PPV), negative predictive value (NPV), and accuracy of p300 where sensitivity is the probability that a test result will be positive when the disease is present, specificity is the probability that a test result will be negative when the disease is not present, and positive predictive value is the probability that the disease is present when the test is positive. Negative predictive value will be negative when the disease is not present. Positive predictive value is the probability that the disease is not present when the test is negative. Accuracy is the overall probability that a patient will be correctly classified.

## Results

The present study included 60 patients in addition to 30 apparently healthy volunteers who served as a control group their age ranged from 20 to 65 years and 70% of them were males. Table 1 shows the demographic characteristics and liver function of the studied groups. The HCV group was subdivided into three groups according to the stage of fibrosis (F0-F1, F2, and F3). As regards MMSE total scores, all of the F0-F1 group (ten patients) had no cognitive impairment (MMSE score 25–30). Fourteen patients (8 in F2 and 6 in F3) had a mild cognitive impairment (total score 20–24) while 19 patients (7 were F2 and 12 were F3) had a moderate cognitive impairment (MMSE total score 11–19). None of the patients had severe cognitive impairment (MMSE total score < 10) as shown in Table 2. In this study, there was significant impairment in digit span, attention, concentration, and memory between patients and control with more impairment in the F3 group (Table 3). The frequency of delayed P300 peak latency was significantly increased in the three groups of chronic hepatitis than controls (*P* = 0.029). Meanwhile, the frequency of delay was increased with increasing the stage of fibrosis (Table 4). The mean of the p300 peak latency showed more significant delay in F2 (279 ± 52.2) and F3 (329.3 ± 67.8), than the control (251.2 ± 32.6) [*p* = 0.0157, *p* = 0.000 respectively], while F0-F1 (278.1 ± 65.1) showed delay in latency of P300 than control, but it was statistically not significant (*p* = 0.0919) (Table 5). As regards the p300 amplitude, the F2 and F3 groups had statistically significant decrease than the control with *p* values (0.045 and 0.0001) respectively while F0-F1 had a reduction in amplitude of P300 which was statistically not significant (Table 6). The reduction of the p300 amplitude was significantly increased with increasing the stage of fibrosis. Table 7 shows that all patients with cognitive impairment (reduced MMSE) had abnormal (delayed) peak p300-evoked response while there was a significant increase in the frequency of delayed p300-evoked response among patients with normal MMSE scores with *p* value (0.036). P300 had sensitivity (100%), specificity (59.26), positive predictive value (75%), negative predictive value (100%), and accuracy (81.67) in the detection of neurocognitive impairment in HCV patient (Table 8).

**Table 1:** Demographic characteristics and liver function of the studied groups

Variable	Control	HCV
Age	43.0 ± 9.7	45.3 ± 9.7
Education	12.4 ± 2.2	12.9 ± 2.3
AST	21.6 ± 8.3	56.5 ± 5.1
ALT	22.5 ± 9	57.1 ± 6.1
Total bilirubin	0.8 ± 0.2	1.51 ± 0.2
Albumin	4.1 ± 0.4	4.3 ± 0.5
PT	0.9 ± 0.4	1.1 ± 0.05
PC	95 ± 8.2	93.3 ± 9.2

**Table 2:** Distribution of MMSE scores among the studied groups

MMSE score	control		Chronic hepatitis						*P* value
			F0-F1		F2		F3		
	*N*	%	*N*	%	*N*	%	*N*	%	
11–19	–	0	–	0	7	26.92	12	50	0.000
20–24	–	0	–	0	8	30.77	6	25	
25–30	30	100	10	100	11	42.31	6	25	
Total	30	100	10	100	26	100	24	100	

*MMSE* Mini Mental State Examination

**Table 3:** Comparison between patients with HCV and controls as regards MoCA and MMSE

Mo CA	Controls (*N* = 30)	Chronic hepatitis			*P*
		F0-F1 (*N* = 10)	F2 (*N* = 26)	F3 (*N* = 24)	
	Mean ± SD	Mean ± SD	Mean ± SD	Mean ± SD	
Digit span	1.88 ± 0.67	1.69 ± 0.5	1.57 ± 0.67	1.4 ± 0.4	0.031
Attention	3.47 ± 0.42	3.22 ± 0.31	2.7 ± 1.06	2.1 ± 0.1	0.000
Concentration	1.4 ± 0.05	1.1 ± 0.07	0.91 ± 0.22	0.66 ± 0.12	0.000
Memory	3.68 ± 0.73	3.50 ± 0.53	3.22 ± 0.76	3.18 ± 0.55	0.026
Orientation	5.7 ± 0.37	5.6 ± 0.50	5.5 ± .73	5.3 ± 0.53	0.071
Moca total score	26.34 ± 1.41	23.2 ± 4.1	22.7 ± 4.2	20.7 ± 2.8	0.000
MMSE	27.53 ± 1.71	26.33 ± 1.3	22.85 ± 4.44	20.17 ± 4.86	0.000

*MoCA* Montreal cognitive assessment, *SD* standard deviation, *MMSE* Mini Mental State Examination

**Table 4:** Frequency of abnormal P300 peak-evoked potential latency between HCV patients and control groups

P300 latency	Controls *N* = 30	Chronic hepatitis			*P*
		F0-F1 *N* = 10	F2 *N* = 26	F3 *N* = 24	
Normal	30 (100%)	6 (60%)	6 (23.08%)	4 (16.7%)	0.029
Abnormal	–	4 (40%)	20 (76.92%)	20 (83.3%)

**Table 5:** Comparison of the P300-evoked potential latency between the studied groups

P300 latency (ms)	Control *N* = 30	Chronic hepatitis			P4
		F0-F1 *N* = 10	F2 *N* = 26	F3 *N* = 24	
Mean ± SD	251.2 ± 32.6	278.1 ± 65.1	279.8 ± 52.2	329.3 ± 67.8	
*P* value		0.0919	0.0157	0.0001	0.000

P1 = control vs. F0-F1, P2 = control vs. F2, P3 = control vs. F3, and P4 is the significant difference between the 4 groups

**Table 6:** Comparison between the studied groups regarding P300 amplitude

P300 amplitude (uv)	Controls *N* = 30	Chronic hepatitis			P4
		F0-F1 *N* = 10	F2 *N* = 26	F3 *N* = 24	
Mean ± SD	13.4 ± 4.02	13.3 ± 4.2	11.1 ± 4.2	9.5 ± 3.4	
*P* value		0.9466	0.0456	0.000	0.002

P1 = control vs. F0-F1, P2 = control vs. F2, P3 = control vs. F3; P4 is the significant difference between 4 groups

**Table 7:** Comparison of MMSE and peak latency of p300 percentage among the studied groups

Groups	N	MMSE	P300		*P*
			Normal	Abnormal	
F0-F1	10	Average 10 Deficient 0	6 (20%)	4 (80%)	0.036
F2	26	Average 11 Deficient 15	6 (23.08%)	5 (19.23%) 15 (57.96%)	
F3	24	Average 6 Deficient 18	4 (16.67%)	2 (8.33%) 18 (75%)

**Table 8:** Sensitivity, Specificity, PPV, NPV, and accuracy of P300 in the detection of neurocognitive impairment in HCV group

Sensitivity	Specificity	PPV	NPV	accuracy
100%	59.26%	75%	100%	81.67%

*PPV* positive predictive value, *NPV* negative predictive value

## Discussion

Cognitive dysfunction in patients with chronic hepatitis C virus (HCV) infection is a distinct form of minimal hepatic encephalopathy (MHE). In fact, the majority of HCV-positive patients, irrespective of the grading of liver fibrosis, display alterations of verbal learning, attention, executive function, and memory when they are evaluated by suitable neuropsychological tests. Similarities between the cognitive dysfunction of HCV patients and MHE of patients with different etiologies are unclear [2, 13, 14]. HCV is neurovirulent that may cross the blood–brain barrier via infected monocytes using a Trojan horse mechanism [3]. Microglial cells and, to a lesser extent, astrocytes harbored HCV-RNA sequences and HCV-specific proteins [15]. HCV replicates in the brain and plays a role in the pathogenesis of neuroinflammation [16] with the increase in the proinflammatory cytokines in the brain. There is also an increase in the choline/creatine and myo-inositol/creatine ratios in the basal ganglia. *N*-Acetyl aspartate (NNA) and NNA-glutamate are raised in the basal ganglia and prefrontal cortex. These metabolites are raised as a compensatory mechanism to HCV inflammation. When these compensatory mechanisms fail, fatigue and possibly other signs of neurocognitive impairment take place [17–20]. Neuroimaging studies suggest that HCV-positive individuals show altered structure and function of several neuronal systems, including the frontal neocortex, basal ganglia, and connecting white matter tracts [3].

In the present study, the F0-F1 group had no cognitive impairment (MMSE score 25–30). Fourteen patients had mild cognitive impairment (MMSE total score 20–24), eight of them in the F2 group and six in the F3 group; 19 patients had moderate cognitive impairment (MMSE total score 11–19), seven of them in the F2 group and 12 in the F3 group, and none had severe cognitive impairment. There was also a significant difference in impairment in digit span, attention, concentration, and memory between patients and control which increased with increasing the grade of fibrosis evidenced by MoCA and MMSE. In agreement with these results, Hamdy et al. [21] found that most of his accidentally discovered HCV patients showed mild cognitive impairment, which was higher in dominant hemisphere functions: attention, naming, memory, fluency, abstraction, and orientation. However, their patients were accidentally discovered blood donors and recently diagnosed that explain the mild impairment. Hilsabeck et al., [5] who studied 80 patients, also found that HCV patients had a significant deficits in attention, learning, psychomotor speed, and mental flexibility and reported that greater impairment was associated with greater fibrosis stage. Similarly, Forton et al. [22] who evaluated the cognitive performances of 43 HCV patients found that HCV patients had worse concentration and psychomotor speed. Huckans et al. [23] also found that HCV patients have a lower score in memory, recognition, and attention tests than those without the infection. In a different study which was evaluating the impact of HCV infection on cognitive functions in children with normal liver function tests, Abu Faddan et al. [24] found that HCV-infected patients were impaired in more cognitive tasks than healthy volunteers. The most significant differences found on measures of concentration and information processing speed. In contrast, Cordoba et al. [25] and Soogoor et al. [26] both reported no correlation between HCV and cognition (very young subjects); however, for Cordoba, patients were accidentally discovered. Similarly, Abrantes et al. [27] found no evidence of an association between HCV infection and cognitive impairment. However, the small number of subjects examined in the last study may have affected result interpretation.

In our study, we also detected the cognitive function by p300 method. We found that the frequency of delayed P300 peak latency was increased in the three groups of chronic hepatitis than controls (*P* = 0.029) and that the delay of P300 peak latency and reduction of P300 amplitude were significantly evident in F2 and F3 groups than control group while F0-F1 showed delay in latency of P300 and reduction in amplitude of P300 than control but they were statistically not significant. The prolonged latency and amplitude reduction of P300-evoked responses were significantly increased with increasing the stage of fibrosis. These results were agreed with that of Kramer [2] who studied 100 patients (25 cirrhotic); he found that HCV individuals had P300 delayed latencies and reduced amplitudes. He also reported that abnormal P300 characteristics were not related to the degree of histological or biochemical activity of hepatitis. In another study [28] involving patients with cirrhotic as well as non-cirrhotic liver disease, P300 latencies have been reported to be similar in non-encephalopathic patients as well as the control, thereby suggesting that P300 latencies were not sensitive in detection of subacute hepatic encephalopathy. Moreover, some investigators, Amido et al. [29] and Senzolo et al. [30], reported that P300 was not useful in detecting cerebral function alterations in cirrhotic patients with no apparent encephalopathy. In this study, the percentage of abnormal p300 peak latency in studied patients, which was significantly greater than those of cognitive impairment (measured by MMSE).In our study, we detected the sensitivity, specificity, PPV, NPV, and accuracy of p300. We found that the p300 is a sensitive and accurate method in detecting early changes in the brain function of patients with chronic hepatitis (sensitivity 100%, specificity 59.26%, positive predictive value 75%, negative predictive value 100% and accuracy 81.67%). Ciećko-Michalska [31] suggested that ERPs are a more sensitive method than psychometric tests in detecting early changes in the brain function of patients with cirrhosis. In partial agreement, Jones et al. [32] had also found in the visual ERP study that the prolongation of P300 latency in cirrhotic patients without overt hepatic encephalopathy (HE) may occur in the absence of abnormalities of a standard psychometric test.

## Conclusions

Patients with CHC infection has significant impairment of digit span, attention, concentration and memory and this cognitive impairment increases with severity of hepatic fibrosis. Patients without fibrosis show no cognitive impairment by psychometric tests while showing impairment by event-related potential. P300 can detect minimal and subclinical impairment of cognitive function at early stages of chronic hepatitis (F0) with a sensitivity 100% and accuracy 81.67% even with normal standard psychometric tests. A future study is needed with larger F0 chronic hepatitis patients to confirm this suggestion.

## Abbreviations

(*χ*^2^): Chi-square test
ANOVA: Analysis of variance
CHC: Chronic hepatitis c
ERPs: Event-related potentials square
HCV: Hepatitis C virus
HE: Hepatic encephalopathy
HIV: Human immunodeficiency virus
MHE: Minimal hepatic encephalopathy
MMSE: Mini-Mental State Examination
MoCA: Montreal cognitive assessment scores
PCR: Polymerase chain reaction
SD: Standard deviation
